# Medical Items

**Published:** 1917-01

**Authors:** 


### MEDICAL ITEMS

### BASE HOSPITAL OFFERED RED CROSS AS MEMORIAL.

Eli Lilly & Company of Indianapolis has offered the local chapter of the American Red Cross, $25 000, in event of this country being drawn into war, to establish a base hospital of establish a base hospital of 500 beds, surgical and medical equipment, and tentage.

The offer was made as a memorial to Colonel Eli Lilly whose splendid service as a soldier and a citizen is worthy of the highest honor that can be accorded him.

The equipment of this base hospital will include every kind of supplies from surgical instruments to bandages and clothing for patients. It is the intention of the local medical society to form a staff to operate the Colonel Lilly Memorial Hospital, which will be made up of twenty-three medical officers, two dentists, a chaplain, fifty nurses, twenty-five nurses' aids, fifty men for administrative duties, and ten civilians for general assignments. In event of war the unit staff will be available to move forward with the equipment of the hospital. Steps have already been taken to organize classes of men and women for training in first aid. The entire equipment and staff will be under the command of the Red Cross.

---

### IN CHILDREN AND IN OLD PEOPLE.

Kidneys are often affected by exposure to cold or chill. These disturbances may range from sudden and frequent desire to the severe forms of urinary irritation. The first is usually accompanied with free and excessive flow of water, where in the latter case there will be but a small quantity of water, frequently passed with difficulty and pain. If the cause is not removed, this disuria with frequency may continue day and night until cystitis occurs, or until a spastic renal condition is found to be present with active congestion follow quickly by acute inflammation. The remedy is heat persistently applied externally to produce relaxation, and sanmetta in drachm doses for adults every hour until relief, then less often as indicated, and half doses for child in like manner. Particularly is it true with men suffering from prastatic trouble that they are often affected by exposure to cold or chill, causing congestion at the bladder neck, with frequent desire to urinate, and urine passed with difficulty and pain. Hot applications externally, either moist or dry, and sanmetto in teaspoonful roses every hour until relief, is the remedy.

### VEST POCKET REFERENCE BOOK ON VACCINE AND SERUM THERAPY SENT PHYSICIANS ON REQUEST.

What physician would not welcome a convenient Vest Pocket Reference Book on Antitoxins, Serums, and Vaccines; (Bacterins)? A Compendium of this kind is being distributed to physicians, without charge, by Eli Lilly & Company. It contains, in addition to a priced list of biological products, brief instructive sections on Infective and Infectious Diseases, the Development of Immunity, Specific Therapy, Serums, and Antitoxins, Anaphylaxis, Indications, Dosage, and Keeking Qualities of Biologicals The Book is thoroughly cross indexed and designed to supply answers to practical questions that arise daily in the administration of biological products.

This handy manual was prepared for free distribution and will be found a convenient vest pocket reference work. It is printed on thin paper, occupies little space, and contains much information of value on vaccine and serum therapy. A copy will be sent without charge to readers of this journal on request made to Eli Lilly & Company.

---

### AN ESTABLISHED FACT.

If there is one therapeutic fact that has been established beyond all question, it is that intestinal indigestion traceable to hepatic torpor and insufficient biliary secretions, will quickly respond to the action of Chionia.

This reliable preparation of Chionanthus Virginica is especially effective in these cases since it promptly stimulates the liver and produces a notable increase in the biliary output, without setting up pronounced catharsis. Thus it not only controls intestinal fermentation, but augments the secretory activity of the intestinal glands. As a consequence, the severe pain that so often characterizes intestinal indigestion—coming on two or more hours after the injestion of food—is quickly controlled; the sallow, muddy complexion due to the absorption of intestinal poisons rapidly clears up; and the patient's whole condition shows substantial improvement.

